# Costs of cervical cancer screening and treatment using visual inspection with acetic acid (VIA) and cryotherapy in Ghana: the importance of scale

**DOI:** 10.1111/j.1365-3156.2010.02722.x

**Published:** 2011-01-09

**Authors:** Wilm Quentin, Yaw Adu-Sarkodie, Fern Terris-Prestholt, Rosa Legood, Baafuor K Opoku, Philippe Mayaud

**Affiliations:** 1 Department of Health Care Management, Technische UniversitätBerlin, Germany; 2 School of Medical Sciences, Kwame Nkrumah University of Science and TechnologyKumasi, Ghana; 3 London School of Hygiene & Tropical MedicineLondon, UK

**Keywords:** economics, costs and cost analysis, uterine cervical neoplasms, Africa, early detection of cancer

## Abstract

**Objectives:**

To estimate the incremental costs of visual inspection with acetic acid (VIA) and cryotherapy at cervical cancer screening facilities in Ghana; to explore determinants of costs through modelling; and to estimate national scale-up and annual programme costs.

**Methods:**

Resource-use data were collected at four out of six active VIA screening centres, and unit costs were ascertained to estimate the costs per woman of VIA and cryotherapy. Modelling and sensitivity analysis were used to explore the influence of observed differences between screening facilities on estimated costs and to calculate national costs.

**Results:**

Incremental economic costs per woman screened with VIA ranged from 4.93 US$ to 14.75 US$, and costs of cryotherapy were between 47.26 US$ and 84.48 US$ at surveyed facilities. Under base case assumptions, our model estimated the costs of VIA to be 6.12 US$ per woman and those of cryotherapy to be 27.96 US$. Sensitivity analysis showed that the number of women screened per provider and treated per facility was the most important determinants of costs. National annual programme costs were estimated to be between 0.6 and 4.0 million US$ depending on assumed coverage and adopted screening strategy.

**Conclusion:**

When choosing between different cervical cancer prevention strategies, the feasibility of increasing uptake to achieve economies of scale should be a major concern.

## Introduction

Cervical cancer is the second most frequent cancer in women worldwide and is responsible for an estimated 493 000 cases and 274 000 deaths annually (Parkin *et al.* 2005). More than 80% of cervical cancers occur in developing countries (Parkin & Bray 2006), and in Ghana, as in sub-Saharan Africa more generally, cervical cancer is the leading cause of female cancer deaths (Shibuya *et al.* 2002; Parkin *et al.* 2005; WHO/ICO 2007). Human papillomaviruses (HPV) are the aetiological agents of cervical cancers (Walboomers *et al.* 1999), and vaccines against HPV are promising for future primary prevention of the disease (Goldie *et al.* 2008; Bonanni *et al.* 2009). However, currently, vaccination is beyond the financial reach of many developing countries, and therefore, improving screening coverage remains central to achieving reductions in female cancer mortality in the short term.

In developing countries, logistical barriers in implementing screening programmes using cytology on Papanicolaou-stained cervical smears to detect precursor cervical lesions have led to failures in reducing cervical cancer incidence and mortality (Denny *et al.* 2006; Gakidou *et al.* 2008). Alternative strategies based on visual inspection of the cervix with acetic acid (VIA) or Lugol’s Iodine (VILI) increase the feasibility of screening in resource-poor settings (Sankaranarayanan *et al.* 2005; FIGO 2009). These strategies require little laboratory infrastructure and provide immediate or very rapid results, allowing treatment with cryotherapy where required in the same visit (Denny *et al.* 2005).

The performance of VIA in routine practice has been assessed in a series of large-scale studies (Sankaranarayanan *et al.* 2004; Sangwa-Lugoma *et al.* 2006; Cuzick *et al.* 2008), and reductions in cancer mortality have been observed in a large Indian screening trial linking VIA with cryotherapy in a single-visit approach (Sankaranarayanan *et al.* 2007). Today, VIA followed by immediate cryotherapy for all eligible women is one of the internationally recommended screening strategies for developing countries (FIGO 2009; Sherris *et al.* 2009), even though new low-cost rapid HPV DNA detection tests (e.g. *Care*HPV™) that have shown higher sensitivity and specificity than VIA (Qiao *et al.* 2008) are about to become available.

In Ghana, where the lifetime risk for women of developing cervical cancer is about 2.2%, and where 2000 women are estimated to develop cervical cancer every year (WHO/ICO 2007), VIA was introduced in several pilot sites in 2001 (Blumenthal *et al.* 2007; JHPIEGO 2008; Sanghvi *et al.* 2008). Subsequently, the feasibility, safety and acceptability of VIA combined with cryotherapy for treatment of precancerous lesions in Ghana was confirmed (Blumenthal *et al.* 2007), and plans for scale-up of a national VIA-based screening programme were developed (Odoi-Agyarko 2003). While lack of a strong political will and competing health priorities may have prevented the programme from being implemented (Sanghvi *et al.* 2008), a new project organized around Kintampo rural health training centre has recently trained providers in an attempt to scale-up VIA screening in rural areas.

International studies have shown VIA combined with cryotherapy to be more cost-effective than traditional cytology-based screening strategies (Goldie *et al.* 2001, 2005; Mandelblatt *et al.* 2002; Legood *et al.* 2005). However, these studies did not base their calculations on directly observed costs, but relied on fee schedules or a series of assumptions and expert opinions, except for the study by Legood *et al.* (2005) which collected data during a large screening trial in India. Yet, to plan for scale-up of screening programmes and to estimate cost-effectiveness of VIA combined with cryotherapy, policy-makers and researchers need reliable estimates based on local costs.

This study was conducted in July and August 2009 to obtain information on the costs of VIA and cryotherapy in an African setting. Specifically, it aimed to (i) estimate the costs of screening using VIA and treatment with cryotherapy at existing screening facilities in Ghana; (ii) explore the most important determinants of costs through modelling and (iii) estimate national scale-up and annual programme costs under varying assumptions for the level of coverage and the frequency of screening.

## Methods

### Cost analysis of VIA and cryotherapy at screening facilities in Ghana

Incremental economic costs of VIA and cryotherapy were estimated in two steps using an ingredients approach: (i) quantities of resources used were measured, and (ii) unit costs or prices were assigned to resources consumed (Drummond *et al.* 2005). The analysis adopted a provider perspective, including only costs of delivering the service while excluding costs incurred by private households or administrative costs at higher levels of the health system (Creese & Parker 1994). As VIA usually constitutes only a small part of activities at existing facilities, we assumed that no additional administrative overheads (such as for hospital administrators or supplies managers) would be incurred and excluded these from consideration.

Data on resource requirements were collected at four of the six active VIA screening facilities in Ghana – three in the Kumasi area (Komfo Anokye Teaching Hospital, Kumasi South Hospital and Sepe Dote clinic), and one in Accra (Ridge Hospital). Providers were observed during performance of their regular activities for between one and 3 days per facility. Providers generally followed the VIA and cryotherapy protocol that had been established during the initial set-up of the VIA pilot-study in Ghana (Blumenthal *et al.* 2007; JHPIEGO 2008). However, while the initial protocol had envisaged VIA screening and treatment with cryotherapy only for women aged 25–45, providers had started to screen also older women if the squamocolumnar junction was visible.

Information was collected on capital (building, equipment) and recurrent (personnel, supplies) resources used for VIA and cryotherapy. For VIA, resource use was collected only for activities directly related to the performance of VIA, i.e. we did not include resources used for evaluation of women referred to physicians for apparent cancer during visual inspection. For cryotherapy, the entire treatment process was included, i.e. extra counselling after a positive VIA test, treatment with cryotherapy, post-treatment counselling and one follow-up visit after 2 weeks.

To estimate unit costs of labour, staff were asked to estimate the percentage of effective working time, which was defined as the time during which they were seeing patients, and the percentage of daily working time dedicated to cervical cancer-related activities. Further information was obtained from actors involved in developing the VIA pilot sites and in attempts to scale-up VIA in rural areas. Resource requirements for mobilization/recruitment of women were estimated on the basis of information on mobilization/recruitment activities at Kumasi South hospital. Costs attributable to maintenance and utilities were calculated based on results of a prior costing study from Ghana (GHS 2000).

Unit costs were collated from multiple sources including the Kumasi University of Science and Technology development office for building construction costs, provider salary slips for personnel costs and market prices for equipment and supplies (a list of unit costs and prices can be obtained from the authors). For personnel, unit costs were calculated by dividing monthly salaries by the estimated effective working time per month. If necessary, costs were adjusted to the year 2009 using the country-specific GDP deflator from IMF (2009) (IMF 2009b). Calculation of annual economic costs of capital items followed recommendations by Tan-Torres Edejer *et al.* (2003) and used a discount rate of 3%. Costs were converted to US$, using the average exchange rate for July 2009 (1 GHS = 0.67 US$). Incremental costs per woman were calculated by multiplying observed resources used per woman with estimated unit costs (see Appendix S1 for further details on data collection and estimation of costs).

### Modelling of costs per woman

A model was constructed to test the influence of observed differences between surveyed facilities and providers on estimated costs. The costing model made assumptions for costs of inputs, number of women screened and treated per provider, effective working time of capital and staff, costs of training and duration of screening/management per woman (Table 1). The model calculated costs for a ‘Base Case Scenario’ using assumed resources used per woman multiplied by their estimated unit costs and tested the influence of alternative assumptions for input parameters through sensitivity analyses (SA).

**Table 1:** Input parameters for modelling of VIA/cryotherapy costs per woman in Ghana

	Basic assumptions	High cost	Base case	Low cost
Capital – buildings				
Size	16 m^2^			
Costs per m^2^ (US$)		2699	1350	1113
Percentage of effective working time		40%	60%	80%
Capital – equipment				
General equipment			International equipment	Locally manufactured equipment
Cryotherapy-specific equipment working-life of Cryogun (years)*		1	2	3
Number of patients per year†		5.1	45	60
Capital – discount rate		5%	3%	0%
Time requirements				
VIA (min)‡		45	17	15
Cryotherapy (min)§		60	50	45
Recurrent – staff				
Doctor salary (US$)		1620	1350	1012
Nurse salary (US$)		750	389	236
Assistant salary (US$)			81	
Number of VIA per nurse per year		1000	600	200
% effective working time¶		40%	60%	80%
Supervision (for 4 nurses)	4 doctor h/month + 1 doctor week per year			
Recurrent supplies				
VIA cryotherapy		With gas for boiling of instruments	Without gas for boiling of instruments	
Recurrent – mobilisation/recruitment (Church group visits (5 nurse h/month + 3.37 US$ transport), central market broadcasts, FM station broadcasts)				
Costs per mobilised woman (US$)			1.50	
Capital – training course and initial supervision				
Trainees	2 doctors, 4 nurses			
Facilitators	2 doctors, 2 nurses			
Duration	2 weeks			
Travel allowance (for trainers)	33.75			
Per diem (trainers) (US$)	33.75			
Per diem (trainees) (US$)	5			
Supervision Year 1	2 doctor h/day			
Supervision Year 2	4 doctor h/week			
Time before retraining**	5 years			

* At Ridge hospital, cryo guns were replaced every year. At Kumasi South hospital: every other year.

† Ridge hospital performed 45 procedures in 2008. Based on an observed VIA positivity rate of 2.53%, assuming 200 screens at low case load facilities would yield about 5.1 positive screens per year.

‡ At Kumasi South hospital: 17 min. At Sepe Dote: 45 min. In Goldie et al. (2001): 15 min.

§ At Kumasi South hospital: 45 min. At Ridge hospital: 60 min.

¶ Estimated effective working time at Kumasi South and Komfo Anokye hospital: 60%.

** Same as in Legood et al. (2005) and supported by Ghana findings.

Base Case assumptions usually reflect findings from observed facilities and considerations about the availability of infrastructure in the country (MOH 2007). High-Cost assumptions are based on observed less efficient providers and building costs at higher quality hospitals. Low-Cost assumptions assume higher efficiency and numbers of screened women, low costs of infrastructure and screening personnel of lower salary categories.

Univariate and multivariate SA were carried out using the alternative ‘High-Cost’ and ‘Low-Cost’ assumptions. For the univariate sensitivity analysis, one model parameter was varied at a time with all other parameters kept constant. For the multivariate SA, all variables were simultaneously set to the ‘Low-Cost’ assumptions or ‘High-Cost’ assumptions, respectively, to generate the largest possible range of costs in an analysis of extremes (Briggs *et al.* 1994).

### Modelling costs of a national VIA/cryotherapy programme

Costs for scale-up to a national screening programme (training of providers, purchasing of cryotherapy equipment, etc.) were estimated using ‘Base Case’ model parameters (Table 1), and assuming a linear relationship between programme inputs and outputs. (Estimated resource requirements for scale-up are presented in natural units in Table S1.) The necessary increase in facility capacity (buildings and equipment) was calculated from the assumed time requirements for VIA and cryotherapy and the number of procedures performed under Base Case assumptions. This yielded an estimated 19% of building space (one room of 16 m square, 60% effective working time) being used for VIA/cryotherapy for every screening nurse employed.

As scale-up costs were estimated separately, national annual VIA/cryotherapy programme costs were calculated for two different scenarios: one scenario excluded start-up costs (training, buildings and equipment); the other scenario included annuitized start-up costs. To do so, modelled incremental economic costs per woman under Base Case assumptions (with/without cost categories of training, buildings, equipment) were multiplied with assumed numbers of women requiring screening in the year 2009 in Ghana. Table 2 presents estimated numbers of women requiring screening under different combinations of assumptions for screening strategy and coverage.

**Table 2:** Estimated number of women in Ghana requiring VIA/cryotherapy in the year 2009

Screening strategy	Screen every five years. Women 25–45 years*	Screen once per lifetime women 35–45 years†
VIA 100% coverage‡	580 406	116 206
VIA 70% coverage	406 284	81 344
Cryotherapy 100% coverage (2.53% of screened)§	15 265	3056
Cryotherapy 70% coverage (2.53% of screened)	10 685	2139
Cryotherapy 100% coverage (10% of screened)¶	58 041	11 621
Cryotherapy 70% coverage (10% of screened)	40 628	8134

* Current practice in Ghana.

† WHO (2002) recommendation for low-income countries introducing cervical cancer screening.

‡ Based on reported female population by age group in WHO/ICO (2007), extrapolated to the year 2009 assuming an average population growth rate of 2.0% (WHO 2006).

§ 2.53% was the proportion of VIA positive women out of all screens between Jan 2008 and July 2009.

¶ According to Sankaranarayanan & Wesley (2003), skilled providers identify 8–15% of screened women as positive. In Blumenthal et al. (2007) test-positivity rate was 13.2%; in Legood et al. (2005): 10%.

## Results

### Costs at surveyed facilities

**Figure 1:**
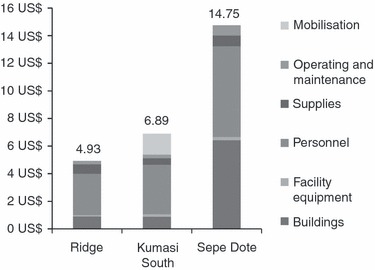
Costs of visual inspection with acetic acid at surveyed facilities: Incremental economic costs per woman (2009 USD).

Figure 1 presents estimated incremental economic costs per woman screened with VIA. Only three of the four visited facilities had VIA cases on survey days. Estimated costs show large variations, ranging from 4.93 US$ (7.30 GHS) to 14.75 US$ (21.86 GHS). Personnel accounted for the largest share of incremental costs at all facilities (between 45% and 61%), while capital costs ranged from 16% to 45%. Costs for mobilization/recruitment per woman were estimated at 1.50 US$ (2.22 GHS), but are included only in reported results for Kumasi South hospital.

**Figure 2:**
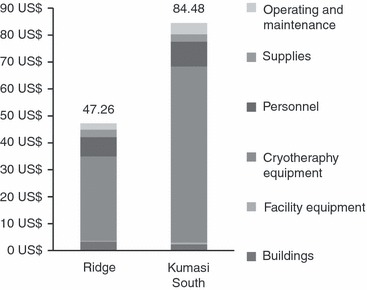
Costs of cryotherapy at surveyed facilities: Incremental economic costs per woman (2009 USD).

Incremental economic costs per woman treated with cryotherapy are presented in Figure 2. Only two facilities had functioning cryotherapy equipment, while the two others were referring women to another hospital for treatment. Reported costs include extra counselling after a positive VIA test, treatment with cryotherapy, post-treatment counselling and one follow-up visit after 2 weeks. The costs per woman treated with cryotherapy in addition to those for VIA varied between 47.26 US$ (70.04 GHS) and 84.48 US$ (125.19 GHS). The cryotherapy equipment accounted for the largest share of costs at both hospitals, comprising 66–77% of total costs.

### Modelled costs per woman

**Figure 3:**
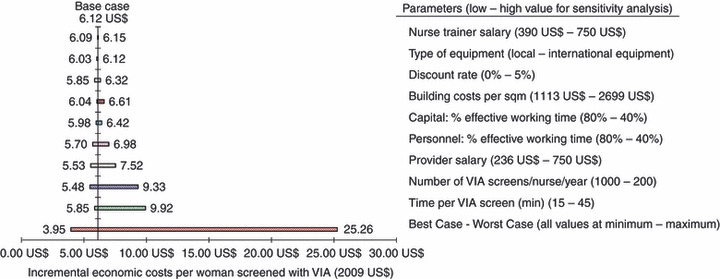
Modelled incremental economic costs per woman screened with inspection with acetic acid and results of sensitivity analysis (SA): Effects of variation of model input parameters on estimated costs.

Figure 3 presents the estimated incremental economic costs per woman screened with VIA. Under base case assumptions for input parameters, VIA costs per woman were estimated at 6.12 US$ (9.07 GHS). Personnel, mobilization/recruitment of women and training of providers were estimated to be the three most important cost categories accounting for 27%, 24% and 23%, respectively. Univariate SA showed that the staff time per VIA screen and the number of women screened per provider have the largest influence on estimated costs per woman. Multivariate SA, simultaneously setting all model input parameters to High-Cost and Low-Cost assumptions, respectively, demonstrated the high impact which alternative assumptions have on estimated costs per woman (lowest bar in Figure 3): the High-Cost Scenario (worst case) estimates are more than four times the size of costs estimated for the Base Case Scenario.

**Figure 4:**
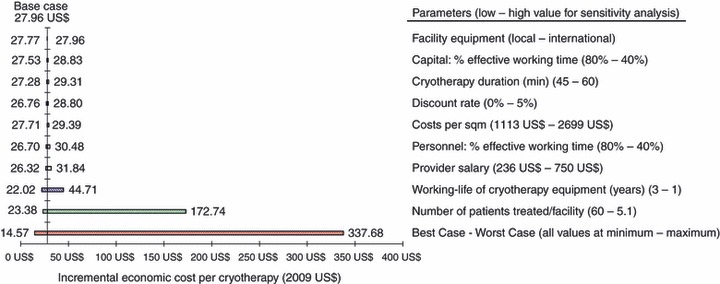
Modelled incremental economic costs per woman treated with cryotherapy and results of sensitivity analysis (SA): Effects of variation of model input parameters on estimated costs.

Figure 4 presents results of modelling the incremental economic costs of cryotherapy under varying assumptions. Under Base Case assumptions for input parameters, incremental economic costs of cryotherapy were estimated at 27.96 US$ (41.43 GHS) per woman treated. Cryotherapy equipment was estimated to account for 62% of total costs. Univariate SA found the number of treated patients per facility per year to be the main determinant of costs of cryotherapy per patient. Multivariate SA showed that costs can increase more than ten times from the Base Case Scenario to about 338 US$ (500 GHS), if all model parameters are set to the alternative High-Cost assumptions.

### National VIA/cryotherapy costs

Table 3 presents estimated VIA/cryotherapy costs for scale-up (investment costs) and annual costs for running a national programme in Ghana. Results are shown separately for different scenarios of coverage and screening strategy and, for annual costs, excluding and including annuitized start-up costs. Depending on the screening strategy and assumed coverage, scale-up (investment) costs were estimated to be between 0.8 and 5.7 million US$ (1.2 and 8.5 million GHS). Accordingly, annual costs for running a national programme (including annuitized costs of capital) were estimated to be between 0.6 and 4.0 million US$ (0.8 and 5.9 million GHS), when assuming a VIA positivity rate of 2.53% as is currently the case in Ghana. Under the assumption of a VIA positivity rate of 10% as reported in the international literature (Sankaranarayanan & Wesley 2003; Legood *et al.* 2005; Blumenthal *et al.* 2007), total costs of the national programme would increase to between 0.7 and 5.2 million US$ (1.1 and 7.7 million GHS) with the proportion of cryotherapy costs rising from 11% (if VIA positivity were 2.53%) to 31% of total screening programme costs.

**Table 3:** Estimated costs for a national VIA/cryotherapy programme: national scale-up and annual programme costs for the year 2009

	Screening strategy			
	
	Every five years		Once a lifetime	
		
	100% coverage	70% coverage	100% coverage	70% coverage
Start-up costs for national VIA/cryotherapy programme (US$)				
Training costs	799 844	559 891	160 141	112 098
Cryotherapy equipment	489 475	342 633	98 000	68 600
Increasing facility capacity	4 444 173	3 110 921	889 789	622 853
Total	5 733 492	4 013 444	1 147 930	803 551
Annual running costs for national VIA/cryotherapy programme (US$)				
VIA	2 367 793	1 657 455	474 067	331 847
Cryotherapy (2.53% of screened)	130 524	91 367	26 133	18 293
Cryotherapy (10% of screened)	515 904	361 133	103 292	72 304
Total (low number of cryotherapy)	2 498 317	1 748 822	500 200	350 140
Total (high number of cryotherapy)	2 883 698	2 018 588	577 359	404 151
Annual costs including annuitized start-up costs (US$)				
VIA	3 554 108	2 487 876	711 585	498 110
Cryotherapy (2.53% of screened)	410 594	287 416	82 207	57 545
Cryotherapy (10% of screened)	1 622 901	1 136 031	324 929	227 450
Total (low number of cryotherapy)	3 964 702	2 775 291	793 792	555 655
Total (high number of cryotherapy)	5 177 009	3 623 906	1 036 514	725 560

## Discussion

A single-visit approach consisting of VIA followed by immediate cryotherapy for all eligible women remains one of the internationally recommended screening strategies for developing countries (FIGO 2009). Our study is the first to report detailed resource-use-based cost estimates for VIA and cryotherapy in an African setting, which are essential for planning of resource allocation and for future cost-effectiveness analyses evaluating VIA against alternative prevention strategies. In addition, we estimated resource requirements for a national cervical cancer screening programme in Ghana.

Results from surveyed facilities showed high variability of VIA and cryotherapy costs in Ghana. VIA costs lie closer to figures previously published for South Africa (10.63 US$) than to those estimated for Kenya (1.31 US$) (Goldie *et al.* 2005) and were also above costs reported for Thailand (1.14 US$) and India (4.68 U$) (Mandelblatt *et al.* 2002; Legood *et al.* 2005).^1^

Modelling of costs per woman found volume effects such as numbers of women screened per nurse or treated per cryotherapy machine to be important determinants. The estimation of national VIA/cryotherapy costs, if such a programme had been in place in Ghana in 2009 showed that under Base Case cost assumptions, total costs of the programme would have remained at <1% of total health expenditures for all combinations of screening strategy and coverage [based on data from 2008 (MOH 2009)].

According to estimates by Goldie *et al.* (2005) for five developing countries, a fully functioning VIA/cryotherapy programme could reduce women’s lifetime risk of cervical cancer by about 50% (assuming sensitivity of VIA of 76%, and a screening strategy with three tests at age 35, 40 and 45 years). However, the effectiveness of VIA has recently come under debate, as most evaluations of the performance of VIA may have overestimated its sensitivity by up to 20% because of use of an inappropriate gold standard (i.e. colposcopy directed biopsy) (Pretorius *et al.* 2007; Cagle *et al.* 2010). Consequently, results of previous cost-effectiveness analyses need to be interpreted with caution, as they assume relatively high sensitivity rates for VIA. In addition, a large-scale 8-year screening trial from India found reductions in advanced cervical cancer incidence and mortality only for women screened with HPV DNA tests and not with VIA or cytology (Sankaranarayanan *et al.* 2009). As more affordable and rapid HPV DNA detection tests (e.g. *Care*HPV™) are becoming available, cost-effectiveness of VIA compared with new HPV tests will need to be reevaluated.

When interpreting our findings, important data and methodological limitations need to be considered. First, some data were not available during our observations of providers’ activities, which make the information less reliable: the percentage of effective working time, resources used for training or for recruitment/mobilization of women and the life-expectancy of cryotherapy equipment had to be estimated based on provider accounts. In addition, providers were unable to relate life-expectancy of cryotherapy equipment to the volume of treated patients. Therefore, our analysis assumed that life-expectancy was not significantly dependent upon the number of patients treated per year. Furthermore, this study was unable to relate costs to relevant outcomes for cervical cancer prevention, as no information was available on the effectiveness of providers in Ghana to detect and treat precancerous lesions. While evaluations comparing providers’ performance quality with that of a ‘Master Trainer’ have confirmed quality was maintained beyond the pilot project (JHPIEGO 2008; Sanghvi *et al.* 2008), VIA positivity rates have dropped from 13% during the time of the pilot project (Blumenthal *et al.* 2007), when quality assurance and regular supervision were still available, to about 2.5% in recent years.

Second, our methodological approach to estimating incremental costs through an ingredients approach may have inherent shortcomings: (i) systems costs (such as administrative and overhead costs) were excluded. Yet, these cost categories are important because insufficient support and attention to management can contribute to programmes failing during implementation; (ii) the ingredients approach may underestimate wastage and losses, which would have been accounted for by using a step down methodology based on total hospital usage (though satisfactory allocation factors for attributing costs of supplies to such a small add-on project remains challenging). Both types of limitations may have led to under-estimation of resource requirements to implement a national cervical cancer screening programme. Furthermore, a national screening programme would result in additional costs for the treatment of detected cervical cancer patients, and for (re)screening of women for whom VIA could not be completed (e.g. if the squamocolumnar junction could not be visualized).

Third, model assumptions are always open to criticism. Our model calculated national programme costs based on assumptions derived from observations at only three screening facilities in the country. In addition, it assumed that mobilization/recruitment costs remained constant for every woman recruited for screening. Yet, it is likely that the costs per woman depend on specific characteristics of the screening programme, such as geographic accessibility of facilities and the duration of the programme. Another limitation is that we calculated national scale-up and annual programme costs under the assumption of constant returns to scale. However, our results suggest the presence of economies of scale in conducting VIA and cryotherapy, which would imply lower average costs per woman screened when screening higher numbers of women. Yet, our sample was too small to identify optimal scale of operation. Furthermore, results would have been different, if alternative High-Cost or Low-Cost estimates would have been considered for calculating annual programme costs.

Despite these caveats, our research has major implications for programme managers and researchers. First, costs of VIA and cryotherapy are highly context-dependent, even within the public sector of one country, showing large variability between facilities. Second, volume effects are an important determinant of costs per woman screened and treated with cryotherapy.

Economies of scale have not been considered in prior cost-effectiveness studies of cervical cancer prevention strategies in developing countries (Goldie *et al.* 2001, 2005; Mandelblatt *et al.* 2002) and are rarely discussed in cost-effectiveness analyses in general (Elbasha & Messonnier 2004). Yet, economies of scale have been found to exist in HIV prevention programmes (Dandona *et al.* 2008; Chandrashekar *et al.* 2010) and are likely to be important for cost-effectiveness analyses informing decisions on different cervical cancer prevention strategies. For example, in a context where low uptake of screening is an impediment to scaling-up, recommendations to screen only once instead of twice or more times per lifetime are likely to result in fewer screenings and, consequently, higher costs per screen. Therefore, international recommendations (WHO 2002; Sherris *et al.* 2009), which were supported by prior cost-effectiveness studies suggesting that screening only once per lifetime is the most cost-effective screening strategy (Goldie *et al.* 2005), may need to be reconsidered.

As scale is an important determinant of costs, policy-makers in Ghana and elsewhere should aim to increase the number of women screened per facility. To do so, demand-side barriers such as user-fees, which were introduced at Ghanaian screening facilities after discontinuation of external support, should be removed and replaced by continued public funding. Availability of services needs to be assured as non-functional cryotherapy machines are discouraging both, providers and clients. Investing in recruitment/mobilization campaigns becomes important to make efficient use of resources. Approaches to integrate screening in family planning clinics appear useful, as they allow easy recruitment of women attending such services, and at the same time, women receiving screening can benefit from treatment of vaginal infections detected during visual inspection.

Policy-makers should be aware that trade-offs may exist: improving geographic accessibility is likely to result in higher costs of screening and treatment per woman. However, concentrating screening at urban centres could exacerbate inequalities between urban and rural populations. Planning for scale-up of cervical cancer screening becomes more difficult when having to consider economies of scale because assuming a simple linear relationship between inputs (employed resources) and outputs (number of women screened/treated) appears to be inadequate. When choosing between different cervical cancer prevention strategies, including vaccination, low-cost HPV tests and VIA/cryotherapy alone or in combination, the feasibility of increasing uptake of screening to achieve economies of scale should be a major concern.

In conclusion, scaling-up screening in developing countries remains essential to achieve reductions in female cancer mortality. In doing so, policy-makers should pay particular attention to the most important determinants of costs of VIA and cryotherapy identified in our study. Research should focus more attention on the determinants of costs and consider that economies of scale are likely to exist when embarking on necessary future cost-effectiveness analyses of alternative cervical cancer prevention strategies.

## Footnotes

1 Published results were converted to year 2009 US$ using (IMF 2009a) GDP deflators and PPP conversion rates.

## Supporting Information

**Appendix S1.** Methodological details of cost analysis.

**Table S1.** Estimated resource requirements for scale-up to a national program.
