# Supplementary material for: Costs of cervical cancer screening and treatment using visual inspection with acetic acid (VIA) and cryotherapy in Ghana: the importance of scale
Source: Trop Med Int Health. 2011 Jan 9;16(3):379–89. doi: 10.1111/j.1365-3156.2010.02722.x (PMC3429861; doi:10.1111/j.1365-3156.2010.02722.x)
Supplement: Supplementary file 1 [file tmi0016-0379-SD1.doc]

# Technical Appendix

– Methodological details of cost analysis –

# Costing approach and adopted perspective

Following existing guidelines for cost analysis in developing countries (UNAIDS 2000;Creese & Parker 1994), our study adopted a provider perspective: Only costs of delivering the service were considered while excluding costs incurred by private households or administrative costs at higher levels of the health system.

Following recommendations by Creese & Parker (1994) for estimation of scale-up costs, we focused on incremental costs of adding VIA and cryotherapy to existing services using an ingredients approach. As VIA usually constitutes only a small part of activities at existing facilities, we assumed that no additional administrative overheads (such as for hospital administrators or supplies managers) would be incurred and excluded these from consideration. However, in order to allow comparison of estimated incremental costs (of adding the service to existing services) with actual costs of providing the service, full costs were imputed by dividing total service costs through the number of screened/treated patients, whenever obtained data allowed estimation of total costs.

Costs were calculated as both, financial and economic costs Creese & Parker (1994). For most inputs, economic costs were assumed to be equivalent to financial costs. However, for capital inputs, economic costs differ from financial costs as they need to consider the opportunity cost of tied up capital Creese & Parker (1994). When nurses donated their time for recruitment/mobilization of women, donated time was valued at the nurses’ normal wage for economic costs.

# Recurrent inputs

Units of recurrent resources (personnel, supplies) used for performance of VIA were recorded during observation of providers. Average personnel time needed per VIA client was calculated, excluding time unrelated to VIA (such as time for treatment of vaginitis/cervicitis). As no women were screened VIA positive during days of facility surveys, no cases of cryotherapy could be observed, and costs had to be estimated based on provider accounts of time and other resource requirements. Personnel was asked to estimate the percentage of effective working time (patient contact time) and the percentage of daily working time dedicated to cervical cancer related activities.

Maintenance and utilities were assumed to be responsible for 5% of total costs of screening and cryotherapy as they have been found to account for 5% of costs of outpatient visits in Ghana (GHS 2000). Costs for recruitment/mobilization of women were estimated based on the Kumasi South hospital midwife’s activities and incurred costs. Estimated costs were divided by the number of screened patients in the year 2008 and included as recruitment/mobilization costs per client at KSH. In addition, these recruitment/mobilization costs per client were included in the cost estimation model described below. Recurrent supervision and quality assurance of provider performance has been inexistent since discontinuation of the pilot project. However, based on accounts of VIA providers, recurrent supervision costs were estimated and also included in modeling of costs per woman (see Table 1 for assumptions).

# Capital inputs

Annual costs of capital inputs were calculated following international guidelines for cost analysis (UNAIDS 2000; Creese & Parker 1994): Replacement costs and working life of capital items were obtained. Annual financial costs were calculated through straight line depreciation. Annual economic costs were calculated using a discount rate of 3% (Tan-Torres Edeje*r et a*l. 2003).

Replacement costs of building space were calculated using average per square meter construction costs and adding 10% for basic furnishing. Replacement costs of most items of equipment were based on market prices. The replacement cost for cryotherapy equipment was based on information obtained from the University of Utah. The working life of equipment was estimated from experience of providers. The working life of furniture was assumed to be 10 years, and that of buildings 20 years Creese & Parker (1994). Staff was asked to provide estimates for effective working time of building space and equipment considering only the amount of time that rooms were used for provision of services to patients.

Training costs were not included in estimates of costs at surveyed facilities since the pilot project that had trained providers had stopped several years ago and data on costs of training were unavailable. However, based on expert and provider estimates for duration of training courses, numbers of trainers and trainees, per diems and travel costs, and necessary supervision during the first two years of practice (see Webtable II), training and supervision costs were calculated when modeling costs per woman.

# Calculation of costs per woman

*Personnel:* Costs per woman were calculated by multiplying observed (or described) time requirements per client with estimated costs per minute of effective working time (patient contact time). For calculation of full costs, personnel costs were allocated between different provider activities based on information obtained during interviews and – if deemed necessary – adjusted by observational data. Then total costs of annual personnel time related to screening or cryotherapy were divided by the total number of screened or treated patients in the year 2008.

*Supplies:* Costs per woman were calculated by multiplying the number of observed or (described) units of inputs with the obtained market prices.

*Capital inputs:* Incremental costs per woman were calculated by multiplying observed (or described) time requirements per client with estimated costs per minute of effective working time of buildings or equipment (patient contact time). Per woman costs for training and cryotherapy equipment were calculated by dividing total costs through the number of women screened and treated per year. Full costs were calculated by allocating total capital costs between different activities and dividing the allocated share of total costs by the number of screened and treated patients in the year 2008. Allocation between different activities had to consider both: the time a specific building or equipment was used for cervical cancer related work; and the percentage of provider time during cervical cancer related hours actually spent on cervical cancer related work, again excluding treatment of vaginitis/cervicitis or counseling for family planning.

Reference List

1. Creese A & Parker D (1994) *Cost analysis in Primary Health Care: a training manual for programme managers.* World Health Organization (WHO), Geneva.

2. GHS (2000) *Costing of hospital services: Evidence for policy and planning.* Ghana Health Service (GHS), Accra.

3. Tan-Torres Edejer T, Baltussen R, Adam T *et al.* (2003) *Making choices in health: WHO guide to cost-effectiveness analysis.* World Health Organization (WHO), Geneva.

4. UNAIDS (2000) *Costing guidelines for HIV prvention strategies.* Joint United Nations Program on HIV/AIDS (UNAIDS), Geneva.
