# Supplementary material for: Costs of cervical cancer screening and treatment using visual inspection with acetic acid (VIA) and cryotherapy in Ghana: the importance of scale
Source: Trop Med Int Health. 2011 Jan 9;16(3):379–89. doi: 10.1111/j.1365-3156.2010.02722.x (PMC3429861; doi:10.1111/j.1365-3156.2010.02722.x)
Supplement: Supplementary file 2 [file tmi0016-0379-SD2.doc]

**Webtable I: Estimated resource require**ments for scale-up to a national program

| **Screening strategy** | **Every five years** | | **Once a life-time** | |
| --- | --- | --- | --- | --- |
|  | 100% coverage | 70% coverage | 100% coverage | 70% coverage |
| **Resource Requirements** | | |  |  |
| Trained personnel (nurses)[[1]](#footnote-2) | 967 | 677 | 194 | 136 |
| Cryoguns and -probes (number)[[2]](#footnote-3) | 326 | 228 | 65 | 46 |
| Increase in facility capacity | 19% of estimated Base Case building and equipment costs for each trained screening nurse | | | |

1. Estimated number of women requiring screening divided by the assumed number of women screened per provider in Base Case Scenario. [↑](#footnote-ref-2)
2. Estimated number of women requiring cryotherapy (assuming VIA positivity of 2.53%) divided by the assumed number of women treated per facility in Base Case Scenario. [↑](#footnote-ref-3)
